# UV “Indices”—What Do They Indicate?

**DOI:** 10.3390/ijerph13101041

**Published:** 2016-10-24

**Authors:** Hanns Moshammer, Stana Simic, Daniela Haluza

**Affiliations:** 1 Institute for Environmental Health, Center for Public Health, Medical University of Vienna, Vienna 1090, Austria; daniela.haluza@meduniwien.ac.at; 2 Institute for Meteorology, University of Natural Resources and Life Sciences, Vienna 1180, Austria; stana.simic@boku.ac.at

**Keywords:** UV indices, weighting function, frequency range, health endpoints

## Abstract

Ultra-Violet (UV) radiation covers the spectrum of wavelengths from 100 to 400 nm. The potency and biological activity for a variety of endpoints differ by wavelength. For monitoring and communication purposes, different UV action spectra have been developed. These spectra use different weighting functions. The action spectrum for erythemal dose is the most widely used one. This erythemal dose per time or dose-rate has been further simplified into a “UV index”. Following this example, in our review we use the term “index” or (plural) “indices” in a more general description for all simplified single-value measures for any biologically effective UV dose, e.g., for human non-melanoma skin cancer and for previtamin D production rate. Ongoing discussion about the existence of an increased melanoma risk due to UV-A exposure underscores the uncertainties inherent in current weighting functions. Thus, we performed an online literature search to review the data basis for these indices, to understand their relevance for an individual, and to assess the applicability of the indices for a range of exposure scenarios. Even for natural (solar) UV, the spectral composition varies spatially and temporally. Artificial UV sources and personal protection introduce further variation to the spectral composition. Many biological effects are proposed for UV radiation. Only few endpoints have been studied sufficiently to estimate a reliable index. Weighting functions for chronic effects and most importantly for cancer endpoints have been developed in animal models, and often for proxy endpoints only. Epidemiological studies on biological effects of UV radiation should not only depend on single-value weighted UV dose estimates (indexes) but should strive for a more detailed description of the individual exposure. A better understanding of the adverse and beneficial effects of UV radiation by wavelength would also improve medical counseling and health communication regarding individual health-supportive behavior.

## 1. Introduction

Epidemiologists love and need simple exposure metrics. Either with a dichotomous parameter (exposed yes/no) or with categorical or continuous variables, modeling is easy so that meaningful risk estimates or even dose-response functions can be established to inform society and policy. However, real life is seldom as straightforward as modeling purposes would expect. In our own ecological study [1] on Austrian data, we have estimated “Ultra-violet (UV) exposure” as a single continuous variable that was mostly influenced by altitude. We found a positive and significant association between exposure and melanoma incidence. Schrempf et al. in this issue [2] correctly point out that the increase in melanoma incidence per 100 m in altitude is much larger than the increase in UV exposure for the same difference in altitude as assessed in our paper. We did not see the same association of altitude or UV exposure with melanoma mortality, and there is some indication that melanoma reporting differs between federal countries in Austria, with more complete reporting also by general practitioners in the more mountainous areas of the provinces of Carinthia and Tyrol.

So there is some evidence that our association between altitude and melanoma incidence might be overestimated. Nevertheless, according to Schrempf et al., our exposure estimates by altitude might be seriously underestimated as well. 

We feel we used the available data in a respectful and considerate way. So why did Schrempf et al. [2] conclude that our exposure estimates were rather biased? We used a weighted metric of UV radiation measured on a horizontal detector. Weighting by frequency was based on the Commission Internationale de l’Éclairage (CIE, International Commission on Illumination) action spectrum for the erythemal dose [3]. While Schrempf et al. point out the problem of the horizontal detector (while, compared to that, on a human body vertical surfaces are also exposed to UV radiation), we want to highlight the fact that we were not really interested in erythema but in melanoma effects. The frequency distribution of the UV spectrum differs by altitude (and, of course, also between direct, reflected and scattered beams) so that an index based on the wrong weighting for the wrong endpoint would give biased results.

We are somewhat relieved to realize that our misconception is encountered quite commonly (just to name a few recent examples, see [4,5,6]). Researchers must use the exposure parameters that are available and the erythemally weighted dose is most commonly provided and hence also used to study UV effects. A simplified version of the erythemally weighted dose rate is termed the “UV index”. Therefore, we use the term “index” (or the plural “indices”) in this paper to describe any simple single-value measure to report the spectrally weighted UV dose for any biological effect.

The intention of the present review article is two-fold: (1) does the spectral efficiency differ by endpoint and is this relevant for effect estimates? Further; (2) how precise and reliable are the currently available indices? We are aware that our paper is not the first to raise the issue. For example, the commentary by de Gruijl [7] in *Radiation Protection Dosimetry* (2000) provides a very fine discussion of the problem and even the abstract is worth reading.

## 2. Materials and Methods

The preliminary literature search examining the current scientific knowledge on estimates for UV-related health effects and UV indices retrieved a limited number of empirical studies. A thorough and systematic literature search on that topic faces many obstacles. Searching all scientific publications using the key words “UV” or “UV radiation” would deliver by far too many papers irrelevant for our questions. On the other hand, a search term like “UV index” would result in a too narrow search and would miss many relevant papers. Indices are usually described in norms and standards and not in the scientific literature. Standards are often costly and since we were not interested in the technical details but rather in the scientific data bases of the weighting functions purchasing all relevant standards was not deemed worthwhile.

We therefore chose systematic snowball sampling for collecting respective publications [8]. We started with the well-known descriptions of the erythemal index [3,9,10] and then sought the references cited therein and also used the “similar citations” option offered by MEDLINE/PubMed services.

Our search began with well-known human health effects, namely erythema, skin cancer, and previtamin D production. Additionally, we soon realized that UV effects on the immune system and on microbes would be of medical interest. We also learned about UV effects on various animal species. We were first interested in that aspect because animals served as model organisms to inform about human health effects. In extension to this, UV effects on terrestrial and aquatic ecosystems are important in their own right. 

All these findings shall be reported here on a “by endpoint” order. We will mainly focus on human health but will also mention non-human effects in a separate subchapter. 

## 3. Results

### 3.1. Definition and Measurement of UV Exposure

The spectral definition of ultraviolet radiation is based on the physiology of the human eye: solar radiation with a wavelength of less than 400 nm is not visible to the human eye and, even at slightly longer wavelengths, the brightness seems to be reduced in comparison to 555 nm at the same irradiance (W/m^2^) [11].

However, there are biological sensors other than the human eye and even different eyes in the animal kingdom differ in their sensitivity towards wavelengths. Even within the human eye rods, the three types of cones capable of color vision, i.e., short-wavelength sensitive, middle-wavelength sensitive and long-wavelength sensitive cones, and various photosensitive retinal ganglion cells display different relative sensitivities to visible light of different color [12]. 

The upper limit of UV light regarding frequency (or the lower limit regarding wavelength) is less well defined, and also the boundaries of the A, B and C bands of UV radiation differ in several definitions. The usual definition, as described by CIE [13] would be UVA 400–315 (or eventually 320) nm; UVB 315 (or 320)–280 nm; and UVC 280–100 nm. Another definition would separate UV radiation into extreme UV (10–100 nm), far UV (100–180 nm), middle UV (180–300 nm), and near UV (300–380 nm) with visible light beginning at 380, not 400 nm. Additional terminology would describe a vacuum UV between 10 and 180 nm and even others speak of “black light” to describe the fuzzy border between visible light and UV radiation (all definitions summarized in [14,15,16]).

For most chemical and, hence, also biological effects, the shorter the wavelength, and therefore the higher the energy per photon, the more effective the UV radiation is. However, there are also specific chemical reactions triggered by UV radiation of a very specific frequency that resonates with the specific electrons involved in the reactive bonds of the chemical structure. Concerning the UV fraction of natural sunlight, mostly the longer wavelengths of UV radiation (UVA) reach the earth’s surface. The fractions with higher energy and hence shorter wavelengths (UVB) are mostly absorbed by the ozone layer and the whole atmosphere. Therefore, not only the intensity, but also the composition of UV radiation differs with altitude.

UV radiation is usually not monitored frequency by frequency, but most detectors measure the whole irradiance in the UV band and use filters to shield off visible light and to approximate the biological activity by frequency [17,18,19]. There are also biological detectors available that inherently capture some biological effects and therefore provide their own weighting function [20,21,22]. Finally, a single number, either an arbitrary index or a weighted irradiance, is reported by each monitor. 

### 3.2. Erythemal Dose and Index

The weighting function for erythema [23] has been proposed by CIE [3,9] and is in widespread use. In this function the normalization wavelength is 290 nm. Between 250 and 300 nm, the relative potency is fairly constant (weighting factor around 1), but it drops dramatically at higher wavelengths. At 330 nm the potency is only about 0.1% of that at 290 nm. Even if other endpoints besides erythema are considered, this weighting function is often applied, e.g., [24]. It is used to estimate “minimal erythemal dose” (MED) by including skin type in the model, e.g., [25]. This approach assumes that the weighting function is independent of skin type or that a darker skin will reduce the penetration of each frequency by the same factor. It is far more likely that skin thickness and pigmentation will affect UV radiation differently depending on frequencies. The “one fits all” weighting function also neglects the facts that “sunburn” is a complex reaction consisting both of inflammatory and phototoxic activities, local and systemic aspects and that even the time course of the reddening of the skin differs by frequency [26]. 

This action spectrum was first proposed by McKinlay and Diffey [27] and the standard weighting function is mostly based on their work but also includes other data [28,29,30,31]. It is thus based on the statistical analysis of many research results. The respective composite curve reflects the average efficiency of the UV of each wavelength in causing erythema and—at least in part—also for melanogenesis [32].

### 3.3. Immunosuppression

De Fabo and Noonan [33] propose an action spectrum for systemic immunosuppression based on animal studies. They suggest that a superficially located photoreceptor is responsible for the observed effects and that this process may also play a role in UV-induced carcinogenesis.

While the authors [33] claimed their spectrum to be related to isomerization of urocanic acid, theirs is not quite identical to the action spectrum for this isomerization [34,35]. Other action spectra were defined that describe local immunosuppressive effects in the skin [36,37]. Effects on the immune system have far-reaching consequences for carcinogenesis and for infection control and therefore must also be considered in this regard (see Section 3.4 and Section 3.6).

### 3.4. Carcinogenesis

Different processes are involved in the development of cancer and different types of cancer are to be considered in the organs exposed to UV radiation. Not surprisingly, different indices or weighting functions have also been proposed to characterize the carcinogenic potency of UV radiation. For example, the action spectrum proposed by Setlow et al. [38,39,40] is based on melanoma in a fish model while Noonan et al. [41] suggested that a transgenic mouse model would be better suited to study these effects and to draw conclusions for humans. 

De Gruij and Van der Leun [42,43] base their estimates of the wavelength dependency of ultraviolet carcinogenesis in humans on mouse data weighted or corrected by differences in epidermal transmission between mice and humans. Their action spectrum is very similar to that for erythema. Indeed, transmission factors would have an important impact on any action spectrum that is relevant for the typical human skin, as most sensitive cells are in the basal layer of the epidermis or even below. Nevertheless, carcinogenesis is a complex process involving many different mechanisms. Genotoxic or mutagenic factors play an important role in the initiation of that process. However, stimulation of cell proliferation, inflammation, and immunosuppression is also important. So it is plausible that cancer (e.g., melanoma) is also induced on parts of the skin that are not exposed to sunlight [44]. A single index would neither capture these differences in UV potency nor would it fit the different types of cancer. Even a combination between (UV) light and chemicals must be considered in the development of certain cancers [45].

### 3.5. Vitamin D

UV radiation also has several beneficial effects on the human body and Ann Webb and co-workers, e.g., [46,47], have provided several seminal papers on that aspect. CIE also provided a technical report and an action spectrum for vitamin D synthesis and for measuring the standard vitamin D dose (SDD) and the minimum vitamin D dose (MDD) [9,48]. The weighting function resembles an inverted “U” with the most effective wavelength around 300 nm. At about 260 nm, the relative potency is 10% of that at 300 nm, while at 330 nm it is only about 0.01%. Different weighting functions were proposed based on in vitro data [49,50,51,52]. These experiments exposed 7-dehydrocholesterol (7-DHC, also called provitamin D3) directly to UV radiation and observed the conversion rate to previtamin D3 or to the active form. As an additional step, the weights were multiplied by the transmission reduction through the stratum corneum, although these reduction factors strongly depend on the color and thickness of the skin under investigation. Norval et al. [53] discuss and compare the validity of the various approaches and suggest that uncertainties remain as to the validity of the “official” spectrum. Seckmayer et al. [54] present a method for calculating vitamin D3-weighted exposure, taking into account all relevant parts of the human body.

### 3.6. Other Effects Relevant to Human Health

UV radiation is also damaging to bacteria [55,56] or fungi [57]. Also, viral infections are affected, although not only by a direct effect on the infectious agents, but also via immunomodulation (see Section 3.3). Direct and indirect effects are therefore numerous and every effect likely is best represented by a somewhat different weighting function. 

Traditionally, spas for treating tuberculosis were placed in mountain areas. One reason for that certainly was the idea of keeping the infectious patients far away from the crowds. However, stronger UV radiation at higher altitudes also helped to prevent the spread of bacteria between patients. Immunomodulation effects and vitamin D deficiency in tuberculosis patients are debated until this day [58].

### 3.7. Ecosystem Effects

Many other weighting functions have been proposed regarding effects on higher plants, marine ecosystems, and for many other endpoints [29,58,59,60,61,62,63,64,65,66]. These are but a few examples to illustrate the diversity of the possible impacts of UV radiation on life on earth. Insofar as they cover maritime ecosystems, the depth of the water column also strongly influences the spectral composition of the UV radiation.

## 4. Discussion

**Figure 1:**
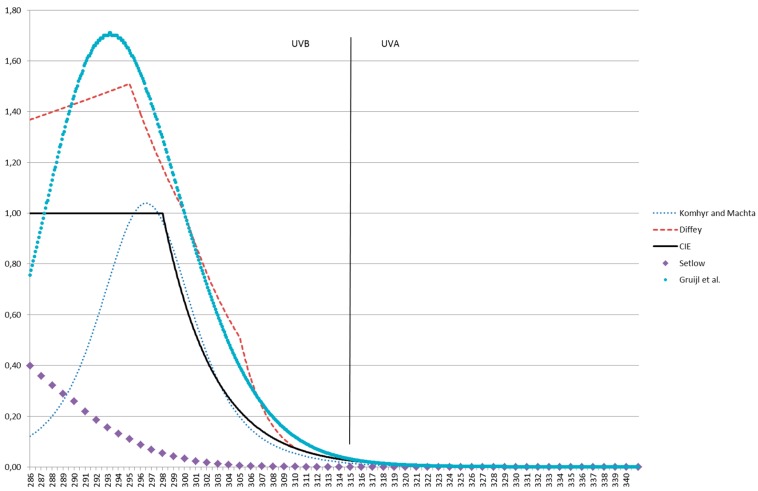
Different weighting functions proposed in the literature. See text for details.

Given the methodological shortcomings of this snowball approach, we cannot claim this to be a comprehensive review. However, we are fairly sure we got a very broad and balanced impression of the topic of interest. In the beginning, we expected one single function for “erythema” (maybe with some variation because of measurement errors or different skin types). We were surprised to learn that even for erythema there is more than one truth. Figure 1 gives a few examples of weighting functions for erythema (Komhyr and Machta [28], Diffey [30], and CIE [3]), as well as one spectrum for DNA damage (Setlow [38]) and one for skin cancer in mice (de Gruijl et al. [43]).

In monitoring networks, UV radiation is usually measured with a spectrometer and exposure in every spectral band is documented separately. This leads to a large amount of exposure data with a strong collinearity between exposures towards the various bands. This is very impractical for epidemiological research, not to mention costs of measurement devices when a comprehensive exposure assessment with high spatial and temporal resolution is sought (e.g., personal monitoring). Moreover, the spectral composition differs strongly between the direct and the reflected and scattered beams. Therefore, the exposure data recorded by a spectrometer also strongly depend on the orientation of its measurement surface. There is no way to ensure that it captures all possible orientations of all surfaces of the human skin [2]. 

Also, because of all these problems, epidemiological research studies often use UV detectors (“dosimeters”) that have an inbuilt weighting function and thus are able to provide a single exposure metric. It even seems a noteworthy feature when such a sensor can be calibrated in two ways to either capture erythemal or vitamin D effective solar ultraviolet radiation [67].

As long as exposures measured by different indices are highly correlated, this would not greatly affect the epidemiological findings. However, when it comes to medical counseling, a wrong quantification of biologically effective UV irradiance can lead to wrong health messages. For example, Kimlin et al. [68] demonstrated that the erythemal dose does not predict the vitamin D effective dose sufficiently well. In winter time at lower latitudes, the message of increasing UV exposure based on erythemal dose is irrelevant and unnecessary and may even lead to excessive exposure.

Epidemiologists usually do not want to study the effect of a certain UV wavelength. Instead, they are interested in some (modifiable) behavior such as “outdoor activity” or “sun bed use”. “Outdoor activity” not only exposes people to UV radiation but also to visible light, infrared, and psychologically relevant sensual stimuli. It also is linked to physical activity and many other relevant factors. How should all these aspects be combined optimally? If the doses of one factor (UV) are only poorly defined in the first place, more complex questions with high public health relevance will not be answered correctly. The power to detect a direct effect might be sufficient even in the face of exposure misclassification. Interaction effects will likely be missed completely.

The situation is even worsened by an ambiguous and sloppy use of terms. Even World Health Organization (WHO) ([15], page 5f) criticizes the inconsistent use of the terms of “minimal erythemal dose” (MED), “standard erythemal dose” (SED) and “solar UV index”. Citing Kollias et al. [69], they conclude: “The lack of a consistent baseline for MED measurement decreases its value for interstudy comparisons.”

While even in natural (solar) UV exposure the spectral composition differs by season, latitude, altitude, and the orientation of the exposed skin (or measurement device), the differences are augmented with individual protective behavior. Sunscreens affect the penetration of the different frequencies differently. Keeping in the shade reduces the UV exposure, but again differently by frequency. Individual differences between texture, color and thickness of the skin, even in the same person at different parts of the body, will also determine effective exposure. 

However, the different spectral effectiveness by endpoint comes into play mostly with technical UV sources. Life on earth has developed under permanent UV exposure at least since it left the oceans. Through evolution, life has partly adapted to UV radiation and has learned to make use of it [70,71]. Technical devices would offer the opportunity to minimize harmful effects and maximize beneficial ones by choosing the optimal spectral composition. But how can we implement that when our effect estimates are based on crude indices’ quantifications only? How can we proceed when experimental research can only assess acute effects while chronic effects are only mirrored by poor proxies? It is sad enough that our concepts of disease are still much too simple. For example, with melanoma, by neglecting differences by subtype, body location, or genes [72], essential risks might either be missed or exaggerated. Also timing, i.e., age at first exposure, must be taken into account [73]. Considering all these complicating factors, it is no surprise that the controversy about the benefits or dangers of sunbeds continues [74,75,76,77].

## 5. Conclusions

On the one hand, simple indices of UV exposure help in the analysis of UV (health) effects. On the other hand, spectral efficiency differs by endpoint and thus a single index can gravely bias the results. Authors should at least be aware of what metric they are using. Often the exposure metric is based on the available monitors and the epidemiologist must make best use of the data available. Whenever possible, frequency-specific exposure data would be preferable, although, because of the collinearity of the various frequency bands, some summarizing effort will be necessary to allow for meaningful analysis. 

Especially for evidence-based health counseling regarding the use of technical devices, a balanced approach calls for a close examination of effects, frequency by frequency. Public health experts should give correct, clear and simple messages. This sentence from the International Commission on Non-Ionizing Radiation Protection (ICNIRP) conclusions [76] maybe serves as an example of how not to phrase a message: “Because of this strong evidence on the adverse health effects of UVR, even though there is not conclusive direct evidence that sunbed exposure causes skin cancer, it is ICNIRP’s view that any use of suntanning appliances is likely to raise the risk of cancer.” Maybe the evidence base has improved since 2003. However, it is our strong conviction that statements would improve when they are based on unambiguous exposure data. The large amount of uncertainty among the general public regarding UV exposure [78,79] underlines this need very strongly.

The epidemiologist studying the health effects of UV exposure should be aware that different biological endpoints are affected differently by different frequencies. If the aim of the study is only to show that there is “some” effect, nearly any weighted dose will do. However, when it comes to describing dose-effect functions and thresholds, it is essential to choose the right weighting function or to keep all the frequency information in the model. Public health officers should also be aware of the benefits and limitations of each “index”.
